# Comparing Ultrasound and Computed Tomography in Regional Anesthesia: Real-Time Precision or Radiologic Clarity?

**DOI:** 10.7759/cureus.87918

**Published:** 2025-07-14

**Authors:** Saba Ahmed, Amudhavan Subramanian, Arzoo Siddiqi, Vignesh Ramachandran, Bushra Kaynat, Malghalara Masood, Sannia Y Dogar, Ali Bilal

**Affiliations:** 1 Anaesthesia and Critical Care, Health Service Executive (HSE) Kerry Community Services, Kerry, IRL; 2 Anaesthesiology, Aneurin Bevan University Health Board, Newport, GBR; 3 Medicine and Surgery, Civil Hospital Karachi, Karachi, PAK; 4 Internal Medicine, Melmaruvathur Adhiparasakthi Institute of Medical Sciences and Research, Melmaruvathur, IND; 5 Anaesthesia, Allama Iqbal Teaching Hospital, Dera Ghazi Khan, PAK; 6 Medicine and Surgery, Bolan Medical College, Quetta, PAK; 7 Medicine and Surgery, Bahria University, Islamabad, PAK; 8 Surgery, Nishtar Medical University, Multan, PAK

**Keywords:** anesthesia, anesthetic spread, clinical outcomes, computed tomography, imaging guidance, interventional pain management, procedural accuracy, regional nerve blocks, ultrasound-guided anesthesia

## Abstract

This systematic review evaluates the comparative effectiveness of ultrasound and computed tomography (CT) in guiding regional anesthesia, with a focus on block accuracy, anesthetic spread, clinical efficacy, and procedural safety. A total of seven high-quality clinical studies were included, encompassing a range of nerve blocks such as celiac plexus, interscalene, intertransverse, and erector spinae plane blocks. Ultrasound guidance consistently demonstrated outcomes that were equivalent or superior to CT guidance in terms of anesthetic distribution, pain control, and patient satisfaction. In most cases, ultrasound enabled real-time visualization, minimized procedural complications, and eliminated radiation exposure while maintaining a high degree of accuracy, as validated by CT or MRI in select trials. CT retained value primarily as a secondary validation tool or in anatomically complex cases where ultrasound imaging is limited. The findings support the preferential use of ultrasound as the first-line imaging modality for regional anesthesia in a variety of clinical contexts. This review highlights the clinical and procedural advantages of ultrasound over CT and emphasizes the need for ongoing research to refine imaging-guided anesthetic techniques.

## Introduction and background

Regional anesthesia plays a pivotal role in modern perioperative care by offering targeted pain relief with reduced systemic complications when compared to general anesthesia [1]. Its growing application across surgical and chronic pain management settings reflects a broader shift toward precision-based analgesic techniques. Central to the success of regional blocks is the accurate identification of neural structures and effective spread of local anesthetic, both of which directly impact clinical outcomes such as analgesic efficacy, block onset, and complication rates [1]. Historically, these procedures were performed using surface anatomical landmarks or peripheral nerve stimulation; however, the introduction of imaging technologies has significantly enhanced the safety, accuracy, and reliability of regional anesthesia [2].

Ultrasound (US) has emerged as a preferred modality for guiding regional anesthesia due to its real-time visualization of nerves, surrounding structures, and needle advancement [3]. It offers several advantages: portability, absence of ionizing radiation, and dynamic assessment of local anesthetic spread. Its use is now standard in many regional block techniques, including brachial plexus, femoral, and paravertebral blocks [4].

Computed tomography (CT), although less commonly used at the bedside, provides high-resolution anatomical imaging and has traditionally been employed in deep plexus blocks, neurolysis, and complex pain management procedures like celiac or hypogastric plexus blocks [5]. CT guidance is particularly useful in anatomically challenging regions where ultrasound may have limited penetration or visualization. However, CT entails radiation exposure and limited real-time capability, making its use more selective [6].

While numerous studies have independently demonstrated the effectiveness of both ultrasound and CT in guiding regional blocks, there remains a lack of consolidated evidence comparing their relative performance in terms of accuracy, anesthetic spread, clinical outcomes, and safety profiles [7]. Given the increasing reliance on image-guided regional techniques, it is critical to evaluate which modality offers superior guidance, especially in complex or high-risk procedures.

The objective of this systematic review is to compare the role of US and CT in regional anesthesia, with a specific focus on block accuracy, anesthetic spread, clinical efficacy, and procedural safety, by analyzing existing clinical trials and imaging studies. This review is structured using the PICO (population, intervention, comparison, outcome) framework [8], where the population includes patients undergoing regional anesthesia for surgical or pain management procedures; the intervention involves ultrasound-guided regional anesthesia; the comparison group consists of patients receiving CT-guided regional anesthesia; and the outcomes assessed include the accuracy of needle placement, the extent and pattern of local anesthetic spread, clinical effectiveness, and complication rates.

## Review

Materials and methods

Search Strategy

A comprehensive and systematic literature search was conducted in accordance with the Preferred Reporting Items for Systematic Reviews and Meta-Analyses (PRISMA) guidelines [9]. The databases searched included PubMed, Embase, Scopus, and the Cochrane Central Register of Controlled Trials (CENTRAL). Additional sources such as ClinicalTrials.gov and Google Scholar were reviewed for gray literature and ongoing trials. Search terms were constructed using Boolean operators and Medical Subject Headings (MeSH) to include variations of keywords such as “ultrasound-guided”, “CT-guided”, “regional anesthesia”, “nerve block”, “computed tomography”, and “local anesthetic spread”. No language restrictions were applied initially; however, only English-language studies were ultimately considered. All references were managed using reference management software to eliminate duplicates.

Eligibility Criteria

Study selection was guided by the PICO framework, where the population (P) included adult patients or healthy volunteers undergoing regional anesthesia; the intervention (I) involved ultrasound-guided nerve blocks; the comparison (C) involved CT-guided nerve blocks or CT used to validate spread; and the outcomes (O) focused on block accuracy, anesthetic spread, clinical efficacy, and patient-reported outcomes. Studies were eligible if they were randomized controlled trials (RCTs), clinical trials, or prospective studies directly comparing US and CT guidance, or using CT/MRI to objectively validate outcomes of US-guided procedures. Inclusion criteria comprised peer-reviewed studies published in English within the last 25 years, involving human subjects, and reporting at least one primary outcome of interest. Exclusion criteria included case reports, animal studies, reviews, editorials, and studies that did not directly assess imaging modality in relation to regional anesthesia outcomes.

Data Extraction

Data extraction was independently performed by two reviewers using a standardized, pre-piloted form to ensure consistency and minimize errors. Extracted variables included study identifiers (author, year), design, sample size, patient population, block type, intervention, and comparator details (e.g., US vs. CT), outcome measures (e.g., pain scores, spread validation, technical success), and key findings. Any discrepancies between the two reviewers were resolved through discussion and, when necessary, adjudicated by a third reviewer. In cases of unclear or missing data, corresponding authors were contacted for clarification.

Data Analysis and Synthesis

Given the heterogeneity in study design, block types, imaging protocols, and outcome measures, a qualitative narrative synthesis was employed. Outcomes were grouped and compared based on imaging modality (ultrasound vs. CT), clinical endpoints (pain relief, satisfaction), anatomical precision (validated by CT or MRI), and adverse effects. Key patterns and discrepancies across studies were highlighted to assess relative efficacy. Formal meta-analysis was not conducted due to variability in outcome measurement and procedural techniques across included studies. The quality and risk of bias of each study were assessed using the Cochrane RoB 2.0 tool [10], and findings were summarized in structured evidence tables to facilitate interpretation.

Results

Study Selection Process

**Figure 1:**
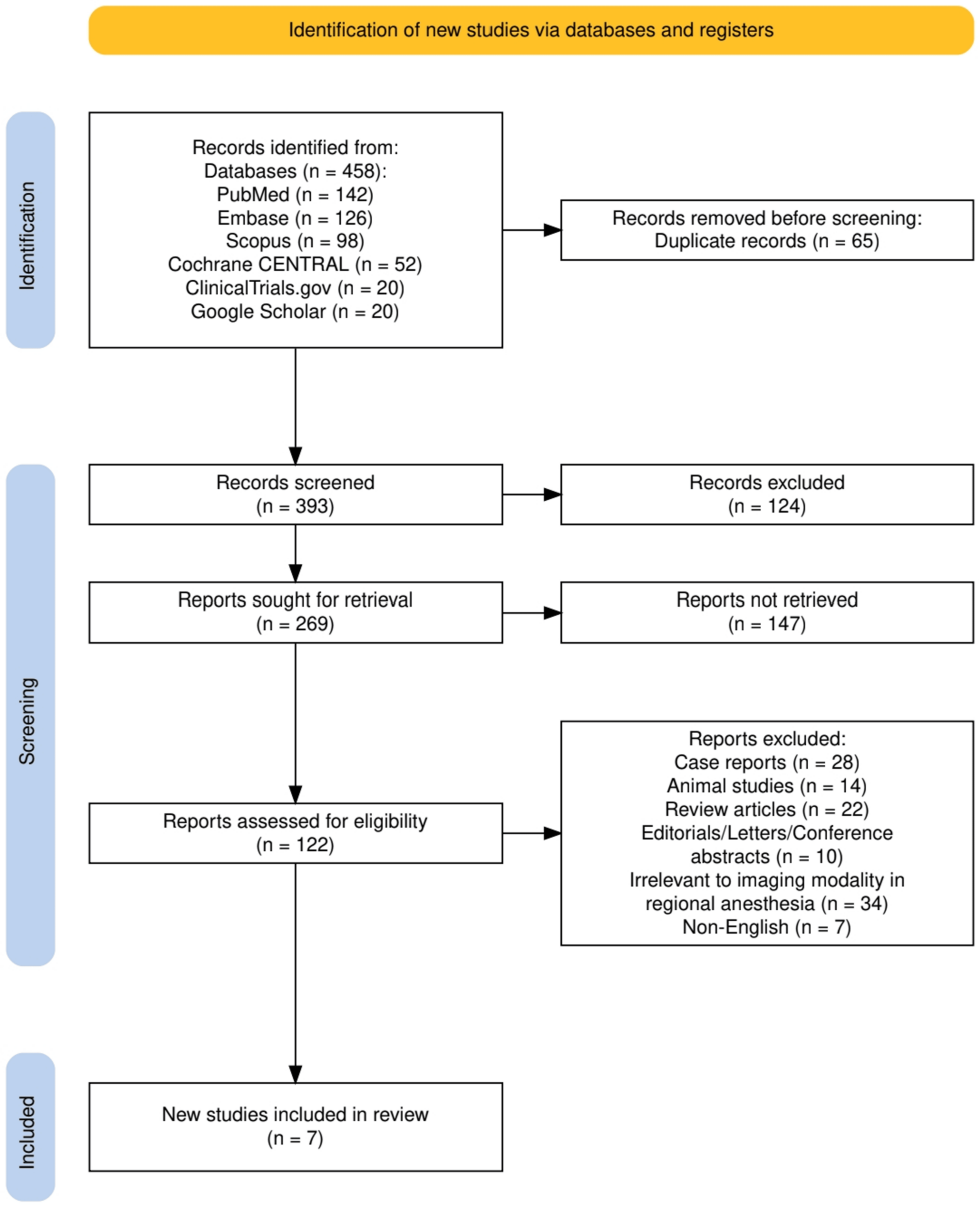
The PRISMA flowchart represents the study selection process. PRISMA: Preferred Reporting Items for Systematic Reviews and Meta-Analyses.

The study selection process adhered to the PRISMA 2020 guidelines and is illustrated in Figure 1. A total of 458 records were identified through comprehensive database and register searches, including PubMed (n = 142), Embase (n = 126), Scopus (n = 98), Cochrane CENTRAL (n = 52), ClinicalTrials.gov (n = 20), and Google Scholar (n = 20). After removing 65 duplicate records, 393 articles remained for initial screening. Of these, 124 were excluded based on titles and abstracts for reasons such as irrelevance or insufficient focus on imaging modalities in regional anesthesia. The full texts of 269 reports were sought for retrieval, but 147 could not be accessed despite best efforts. The remaining 122 full-text articles were assessed for eligibility against predefined PICO-based inclusion and exclusion criteria. Following detailed evaluation, 115 studies were excluded, primarily due to being case reports (n = 28), animal studies (n = 14), review articles (n = 22), editorials or conference abstracts (n = 10), irrelevance to imaging modality (n = 34), or non-English language (n = 7). Ultimately, seven high-quality clinical studies were included in the final synthesis.

Characteristics of the Selected Studies

The key characteristics of the included studies are summarized in Table 1. All seven studies were prospective clinical trials, the majority of which were randomized controlled trials, involving human participants undergoing various regional anesthesia procedures. The population spanned a diverse clinical spectrum, including patients undergoing breast cancer surgery, shoulder arthroplasty, and chronic pain management for pelvic or abdominal malignancies. Ultrasound guidance was used as the primary intervention technique in all studies, with computed tomography either serving as a comparator or as a modality for validating the distribution of anesthetic spread. A wide range of regional block types were represented, including celiac plexus, superior hypogastric plexus, erector spinae plane, interscalene, and intertransverse process blocks. Primary outcomes commonly assessed included the extent and pattern of local anesthetic spread, procedural accuracy, pain scores, and patient satisfaction. Most studies incorporated objective imaging modalities, such as CT or MRI, to quantify spread, enhancing the anatomical validity of their findings.

**Table 1:** Summary of the included clinical studies comparing ultrasound and CT guidance in regional anesthesia. US: ultrasound; CT: computed tomography; RCT: randomized controlled trial; VAS: visual analog scale; ESPB: erector spinae plane block; ITP: intertransverse process; LA: local anesthetic; EUS: endoscopic ultrasound; MRI: magnetic resonance imaging.

Study (author, year)	Study design	Population (n, type)	Procedure/block type	Intervention (US-guided)	Comparison (CT-guided)	Primary outcomes	Key results/findings
Gress et al. (1999) [11]	RCT	18 patients with chronic pancreatitis	Celiac plexus block	EUS-guided injection of bupivacaine + triamcinolone	CT-guided celiac block with the same injectate	Pain relief, reduction in analgesic use, and patient satisfaction	50% relief with EUS vs. 25% with CT; EUS had longer-lasting relief and was preferred by patients
Marcy et al. (2001) [12]	Prospective clinical trial	34 cancer patients with upper abdominal pain	Celiac plexus block with ethanol	Ultrasound-guided (n = 13), real-time color Doppler was used	CT-guided (n = 21)	Technical success rate, pain relief, and feasibility	Pain relief in 79% overall; US success rate 93%, CT 100%; minor complications in 17%; US recommended when feasible
Mishra et al. (2013) [13]	RCT	50 patients with advanced gynecological cancer and pelvic pain	Superior hypogastric plexus neurolysis	Anterior US-guided neurolysis	CT/fluoroscopy-guided (general standard, not active comparator)	Pain scores, morphine consumption, functional capacity, and satisfaction	Greater pain reduction and lower morphine use in the US group; higher satisfaction scores; avoided radiation exposure
Abdella et al. (2022) [14]	RCT	60 breast cancer surgery patients	Erector spinae plane block	US-guided ESPB with 20 ml or 40 ml bupivacaine	CT was used for assessing the spread only	VAS pain scores, LA spread, dermatomal coverage, and satisfaction	Higher volume increased craniocaudal spread (11.2 vs. 9.15 levels); pain scores were better in both ESPB groups vs. control; no added efficacy from more volume
Gautier et al. (2015) [15]	RCT (volunteer study)	9 healthy volunteers	Interscalene space injection	US-guided injection of 10 mL radio-opaque saline under two pressure conditions	CT used post-injection to assess the spread	Injectate spread pattern, root contact, and discomfort	Injectate contacted 3 brachial plexus roots in all volunteers; consistent spread regardless of pressure; validated anatomical coverage via CT
Wang et al. (2025) [16]	RCT	45 adults (18-75 years) undergoing ITP block	Intertransverse process block	US-guided injection of ropivacaine with contrast at 3 volumes	CT was used for assessing LA distribution	Distribution in anatomical compartments, VAS, and sensory loss	LA predominantly spread to paravertebral, visceral, and ESP spaces; volume didn't affect the spread range, position had a significant influence
Stundner et al. (2016) [17]	RCT	30 patients undergoing shoulder surgery	Interscalene plexus block	US-guided with 5 ml or 20 ml ropivacaine + MRI contrast	MRI was used to assess the spread	Epidural and non-epidural spread, phrenic nerve involvement, pain scores, and respiratory function	Epidural spread occurred equally; larger volume caused more phrenic nerve involvement, intervertebral spread, and diaphragmatic impairment; analgesia was similar in both groups

Quality Assessment

The methodological quality of the included studies is presented in Table 2, highlighting the risk of bias across five key domains. Most studies demonstrated low risk in randomization, outcome measurement, data completeness, and selective reporting. In particular, five out of seven studies were assessed as having an overall low risk of bias, owing to robust randomization methods, appropriate blinding, and the use of standardized outcome measures such as validated pain scores and imaging-confirmed anesthetic spread. Two studies were rated as having a moderate risk of bias primarily due to the absence of blinding or lack of randomization, which could introduce performance or detection bias. Nevertheless, all included studies reported outcomes relevant to the review's objectives and showed no evidence of selective reporting, supporting the credibility and reliability of the overall evidence base.

**Table 2:** Risk of bias assessment of the included studies based on the Cochrane RoB 2.0 tool. RoB: risk of bias; RCT: randomized controlled trial; VAS: visual analog scale; CT: computed tomography; MRI: magnetic resonance imaging.

Study (author, year)	Randomization process	Blinding	Outcome data completeness	Outcome measurement	Selective reporting	Overall risk of bias
Gress et al. (1999) [11]	Low – proper randomization reported	Unclear – blinding not mentioned	Low – follow-up done at multiple time points	Low – VAS and satisfaction recorded	Low – outcomes consistent with protocol	Moderate – due to lack of blinding
Marcy et al. (2001) [12]	High – not randomized, observational comparison	High – no blinding	Low – outcomes clearly reported	Low – standard pain relief assessment	Low – no evidence of selective reporting	Moderate – due to lack of blinding
Mishra et al. (2013) [13]	Low – randomized groups with control	Unclear – no mention of blinding	Low – full data on outcomes	Low – pain, function, and satisfaction measured with standard tools	Low – outcomes match study aim	Moderate – due to unclear blinding
Abdella et al. (2022) [14]	Low – clearly randomized and registered	Low – blinding of outcome assessors implied	Low – complete outcome data with stats	Low – validated pain scores, CT analysis	Low – full reporting, registered trial	Low
Gautier et al. (2015) [15]	Low – within-subject design in healthy volunteers	Low – volunteers blinded to pressure levels	Low – all data presented	Low – CT objectively assessed spread	Low – no selective outcome reporting	Low
Wang et al. (2025) [16]	Low – randomized, blinded CT analysis	Low – CT analysis was blinded	Low – full outcomes reported	Low – spread and secondary outcomes clearly measured	Low – published protocol and results align	Low
Stundner et al. (2016) [17]	Low – randomized and blinded operator	Low – outcomes assessed via MRI and blinded personnel	Low – full data, no attrition	Low – standard and imaging-based outcomes	Low – no selective reporting	Low

Discussion

This systematic review aimed to compare the effectiveness of US and CT in guiding regional anesthesia, with a focus on anesthetic spread, procedural accuracy, and clinical outcomes. Seven high-quality studies involving 246 patients (plus healthy volunteers) across multiple block types, including celiac, hypogastric, interscalene, intertransverse, and erector spinae, were analyzed. Ultrasound guidance consistently provided equivalent or superior pain control compared to CT-guided techniques, with faster onset and broader dermatomal coverage in most settings. In Gress et al.'s [11] study, ultrasound achieved a 50% pain relief rate versus 25% for CT at eight weeks. Abdella et al. [14] demonstrated greater craniocaudal spread (11.2 vs. 9.15 vertebral levels) and higher patient satisfaction in US-guided erector spinae plane blocks. In Mishra et al.'s study [13], US-guided neurolysis significantly reduced morphine consumption and improved satisfaction over conventional methods. Additionally, advanced imaging (CT/MRI) used in conjunction with US validated its anatomical accuracy, further affirming its reliability. CT guidance, while effective in specific contexts like retroperitoneal blocks, did not consistently outperform ultrasound in any measured parameter.

Our findings align with the growing body of literature supporting the use of ultrasound as the first-line imaging modality for regional anesthesia [18]. Prior reviews and guidelines from the American Society of Regional Anesthesia (ASRA) and the European Society of Anaesthesiology (ESA) [19] increasingly endorse ultrasound for peripheral nerve blocks due to its real-time visualization, safety, and ease of use. This review reinforces that position, particularly in light of trials such as those by Stundner et al. [17] and Wang et al. [16], which used MRI and CT to confirm precise injectate localization with US guidance. While some early reports favored CT for deeper plexus interventions, newer studies demonstrate that modern high-frequency linear transducers can reach even complex anatomical targets with reduced risk [20]. Our data support this paradigm shift, especially in light of the increased emphasis on radiation-free, patient-centered care.

From a clinical standpoint, the implications are significant. Ultrasound-guided regional anesthesia is not only safer, avoiding radiation exposure, but also logistically favorable due to its portability and real-time guidance [21]. It allows anesthesiologists to dynamically assess anatomical structures and injectate spread, reducing inadvertent vascular puncture or neural injury. CT guidance, while offering high-resolution imaging, is limited by its immobility, radiation exposure, and lack of real-time feedback [22]. Its use may still be justified in deep or retroperitoneal blocks where ultrasound penetration is insufficient or when anatomical distortion precludes clear sonographic visualization. Thus, ultrasound should remain the standard for routine regional blocks, while CT should be reserved for complex interventional pain procedures or research validation [23].

This systematic review is strengthened by its rigorous methodological framework. A clearly defined PICO model guided study selection, ensuring clinical relevance and focus. All included studies were prospective clinical trials or randomized controlled trials, minimizing retrospective bias. The use of objective imaging endpoints, particularly CT and MRI, for confirming local anesthetic distribution added a robust anatomical basis to the clinical outcome data. Moreover, the majority of the studies included in this review were assessed to have low risk of bias, particularly in domains related to randomization, outcome completeness, and objective measurement. This methodological rigor enhances the internal validity and generalizability of our findings.

Nevertheless, several limitations must be acknowledged. The studies included a variety of regional anesthesia techniques, target nerves, and patient populations, introducing procedural and clinical heterogeneity. For instance, the interscalene and intertransverse process blocks differ significantly in technique and anatomical goals compared to celiac or hypogastric plexus neurolysis. Sample sizes in some studies, such as Gautier et al. (2015) [15], with only nine healthy volunteers, limit statistical power and external validity. The trial by Marcy et al. (2001) [12] lacked randomization and blinding, increasing the risk of bias. Moreover, long-term outcomes such as chronic pain development, functional recovery, or cost-effectiveness were rarely reported. In most cases, CT was used as a validation tool rather than a direct comparator, which limits the strength of conclusions regarding head-to-head efficacy.

Future research should prioritize large-scale, multicenter randomized controlled trials that directly compare ultrasound and CT guidance in complex regional anesthesia procedures, particularly in deep-seated or anatomically distorted cases. Economic evaluations should be integrated into study design to assess cost-effectiveness, especially considering the higher capital cost and infrastructure requirements of CT [24]. Research into user proficiency, learning curves, and simulation-based training will be essential to optimize ultrasound technique adoption [25]. Finally, emerging technologies such as fusion imaging, augmented reality, and AI-assisted needle tracking offer promising avenues for enhancing the precision of both ultrasound and CT-guided regional blocks and should be systematically investigated in future studies.

## Conclusions

This systematic review demonstrates that ultrasound-guided regional anesthesia is a safe, effective, and clinically advantageous alternative to CT-guided techniques across a broad range of procedures. Ultrasound not only provides real-time visualization and dynamic control but also avoids radiation exposure and allows for greater portability and procedural flexibility. While CT retains niche value in anatomically complex or deep-seated blocks where sonographic landmarks are obscured, the evidence from this review overwhelmingly supports ultrasound as the preferred first-line imaging modality. These findings hold important implications for clinical practice, resource allocation, and future guideline development in regional anesthetic care.
